# Advances in the treatment of cytomegalovirus

**DOI:** 10.1093/bmb/ldz031

**Published:** 2019-10-03

**Authors:** B A Krishna, M R Wills, J H Sinclair

**Affiliations:** 1 Department of Medicine, Addenbrooke’s Hospital, University of Cambridge, Cambridge, UK; 2 Lerner Research Institute, Cleveland Clinic, Cleveland, OH, USA

**Keywords:** human cytomegalovirus, vaccines, antivirals, infection, latency

## Abstract

**Background:**

Human cytomegalovirus (HCMV) is a threat to immunologically weak patients. HCMV cannot yet be eliminated with a vaccine, despite recent advances.

**Sources of data:**

Sources of data are recently published research papers and reviews about HCMV treatments.

**Areas of agreement:**

Current antivirals target the UL54 DNA polymerase and are limited by nephrotoxicity and viral resistance. Promisingly, letermovir targets the HCMV terminase complex and has been recently approved by the FDA and EMA.

**Areas of controversy:**

Should we screen newborns for HCMV, and use antivirals to treat sensorineural hearing loss after congenital HCMV infection?

**Growing points:**

Growing points are developing drugs against latently infected cells. In addition to small molecule inhibitors, a chemokine-based fusion toxin protein, F49A-FTP, has shown promise in killing both lytically and latently infected cells.

**Areas timely for developing research:**

We need to understand what immune responses are required to control HCMV, and how best to raise these immune responses with a vaccine.

## Human cytomegalovirus establishes an asymptomatic, persistent infection in healthy individuals

Human cytomegalovirus (HCMV) is a large DNA virus of the *Betaherpesvirinae* subfamily, with a double-stranded DNA genome of approximately 230 kb. The HCMV genome is complex and encodes functional proteins, microRNAs, long non-coding RNAs and small peptides.^1^^,^^2^ As is common to all herpesviruses, HCMV persists for the lifetime of the host after primary infection; this persistence is at least partly supported by latent infection, as well as by diverse mechanisms to manipulate and evade the host immune response.^3^

Individuals with healthy immune responses usually show no symptoms after primary infection but on rare occasions can present symptoms similar to infectious mononucleosis.^4^ Very rarely, severe, acute HCMV infections occur in otherwise healthy individuals. These infections most commonly involve symptomatic infection of the gastrointestinal tract, liver and central nervous system; haematological manifestations; and eye, lung or arterial or venous thrombosis.^5^ These patients are treated with antivirals, normally on a case-by-case basis, similar to the immunocompromised, as discussed below.^5^ The reasons for these isolated incidents of severe HCMV infections in immunocompetent people remain unclear.^6^

HCMV infection is usually asymptotic because a robust, healthy immune response controls viremia.^4^ Despite this, HCMV is not cleared from the host but persists by establishing a lifelong latent infection in undifferentiated cells of the myeloid lineage (CD34^+^ haematopoietic progenitor cells and their derivative CD14^+^ monocytes). As these cells exit the bone marrow and differentiate to macrophages and/or dendritic cells, virus reactivation is triggered.^3^ This sporadic reactivation of HCMV from latency in differentiated myeloid cells is also asymptomatic in healthy individuals, but likely helps replenish the reservoir of latently infected cells.^7^ Such sporadic asymptomatic HCMV infection in healthy, seropositive individuals has been linked with an increased incidence of atherosclerosis,^8^ arterial hypertension,^8^ glioblastoma and other cancers (with great controversy as to whether HCMV is oncogenic or oncomodulatory or these observations are simply artifactual)^9^^,^^10^ and Guillain–Barré syndrome,^11^ and such conditions reduce overall life expectancy in these seropositive individuals.^8^^,^^12^

## HCMV can be life-threatening in immunocompromised, immunosuppressed and immunonaïve patients

HCMV primary infection, reinfection with a different circulating HCMV strain and reactivation from latent infection are a serious threat to immunocompromised, immunosuppressed and immunonaïve individuals. The potential for HCMV infection of a many different tissues causes a wide array of potential symptoms, but eventual organ failure may occur if HCMV antivirals are not used as treatment.^13^

## HCMV in neonates

Congenital HCMV infections (cCMVs) are not always controlled by immunonaïve neonates. Infection *in utero* is common (approximately 0.5% of live births^14^), and around 8–10% of infections are symptomatic at birth.^14^ These cases are the most common infectious cause of congenital birth defects, which contributes greatly to the disability-adjusted life-years associated with HCMV^14^ and was estimated to cost the UK £723 million in 2016.^15^ Although the greatest risk of symptomatic congenital HCMV infection is observed upon primary infection of seronegative mothers during the first trimester of pregnancy, the neonates of seropositive mothers are still at risk, due to both reactivation of latent virus or reinfection with a different HCMV strain (known as superinfection).^16^ In December 2017, the UK National Screening Committee did not recommend screening all newborn babies for HCMV using a saliva test,^17^ largely because it is not currently possible to screen for children with HCMV infection that will lead to long-term health problems, and so most babies identified as positive for HCMV will not have any symptoms (https://legacyscreening.phe.org.uk/cytomegalovirus). As it is not clear whether screening will help the outcomes of asymptomatic children, it was not recommended.

cCMV symptoms include cytomegalic inclusion disease (the histopathological detection of inclusion bodies in enlarged, HCMV infected cells), intrauterine growth retardation, jaundice and microcephaly, with developmental delay, sensorineural hearing loss and significant subsequent mortality rates.^14^ Survivors can suffer from multiple disabilities, due to cerebral calcification with neurological, hearing and visual impairments.^18^ The most common neurodevelopmental impairment associated with cCMV is sensorineural hearing loss, and cCMV is responsible for up to 15% of sensorineural hearing loss in the UK.^17^ cCMV in children showing moderate to severe symptomatic disease should be treated with valganciclovir (VGCV, antivirals which will be discussed later) within the first 4 weeks of life for up to 6 months, with monitoring of neutrophil counts and transaminase levels.^15^^,^^19^^,^^20^ Antiviral treatment is not recommended for asymptomatic cCMV, infections with mild symptoms or isolated sensorineural hearing loss, partly due to a lack of randomised control trials providing evidence to support the efficacy of antiviral treatment.^15^^,^^19^ Partly to address this issue, there is currently a randomised controlled trial evaluating benefits of treating older children with confirmed sensorineural hearing loss and cCMV (ClinicalTrials.gov NCT01649869).

## HCMV is a major complicating factor for transplants due to immunosuppression

Immunosuppression after organ transplant leaves patients at risk of HCMV disease.^21^ These include patients undergoing solid organ transplantation as well as bone marrow/haematopoietic stem cell transplants; however, the risk of HCMV-related diseases is different between these groups of patients and risk is dependent on whether the donor, recipient or both are carrying HCMV.

Solid organ transplant (SOT) recipients, who are seronegative (R−) are at the greatest risk of HCMV disease when receiving an organ from a cadaveric, seropositive donor (D+), likely due to both lytic and latent virus being carried in the donated tissue to a patient with no prior anti-HCMV immunity.^22^ HCMV-seropositive recipients have an intermediate risk of disease while the R−/D− combination has the lowest risk (coming only from primary HCMV infections).^23^ Cadaveric organ donations are associated with an increased risk of HCMV disease, most likely due to increased immunosuppression required for cadaveric tissue donation.^24^ As well as HCMV disease, HCMV may be linked to a greater risk of graft rejection, as well as general morbidity and mortality.^25^

Like solid organ recipients, patients receiving allogeneic haematopoietic stem cell transplants (allo-HSCTs) are also at risk of HCMV disease. Umbilical cord blood stem cells, or peripheral blood stem cells, for example from G-CSF-mobilised donors, are now the most common sources of stem cells for allo-HSCT and comprise multipotent CD34^+^ stem cells, which expand and differentiate to reconstitute the immune system. This cellular differentiation can trigger HCMV reactivation and dissemination, as latently infected cells will be transferred if the donor is HCMV-positive.^3^ However, HCMV can also come from primary infection and reactivation of latent viral reservoirs already in an R+ recipient.^26^ Indeed, HCMV reactivation and viral dissemination can occur in up to 80% of allo-HSCT patients if dissemination is not treated with antivirals.^27^

While treatment with HCMV antivirals, blood screening and donor/recipient matching for HCMV serostatus have decreased the incidence of HCMV disease, HCMV still remains a significant threat, especially at later times after transplant.^28^ Matching HCMV serostatus is still the gold standard but is often practicably impossible as it substantially reduces the pool of potential organ donors and can necessitate the co-transfer of T cells (discussed below), which subsequently increases the risk of transplant rejection and HCMV disease. Given this, a vaccine to eradicate HCMV, or treatments that can clear HCMV from infected organs, would clearly have clinical benefits.

## Adoptive transfer of T-cell immunity against HCMV in haematopoietic stem cell transplants

For allo-HSCTs where the donor is seropositive, donor lymphocytes (which will contain HCMV-specific T cells) can also be transferred to help protect allo-HSCT recipients from HCMV infection. These lymphocytes could also play a role in controlling other opportunistic pathogens such as aspergillus.^29^ However, the presence of T cells in the graft also has a disadvantage, as they increase the risk of graft-versus-host disease (GVHD)^30^ and to complicate this, GVHD itself correlates with HCMV disease.^30^

Ideally, the anti-HCMV benefits of T cell adoptive transfer could be gained, without the risk of GVHD, if a clinician could transfer only anti-HCMV donor T cell clones without transferring T cells which would cause the allo-recognition that leads to GVHD. The adoptive transfer of specific anti-HCMV CD8^+^ T cell clones is effective^31^; however, transfer of these cells without helper CD4^+^ T cells means that CD8^+^ clones are short-lived.^31^ Additionally, selecting only a small set of monoclonal anti-HCMV T cells risks the selection of escape mutations so to protect against HCMV, a clinician would ideally transfer broad, polyclonal, anti-HCMV CD4^+^ and CD8^+^ T cells.^32^ Methods to promote the proliferation of anti-HCMV polyclonal T cells from a mixed population of all donor T cells have included using HLA-peptide tetramers, priming PBMCs and dendritic cells with HCMV lysates and transducing dendritic cells with adenoviral vectors expressing the HCMV antigen pp65.^32^ As well as transferring T cells from the D+ stem cell donor, it is also possible to transfer T cells from another D+ donor, which can provide equally effective anti-HCMV protection.^33^

## Ganciclovir is the gold standard treatment for preventing HCMV disease

The current favoured drug treatment for active HCMV infection in the immunocompromised is intravenous ganciclovir (GCV) treatment. GCV is a nucleoside analogue that, when phosphorylated to form ganciclovir triphosphate, preferentially inhibits the viral DNA polymerase UL54.^34^ Initial phosphorylation is catalysed by the HCMV-encoded UL97 kinase, followed by subsequent phosphorylation, catalysed by cellular kinases. As the first phosphorylation step is performed by an HCMV-encoded kinase, this leads to drug selectivity for infected over non-infected cells.^34^ A derivative of GCV is VGCV, a GCV prodrug with oral bioavailability, which is routinely given to SOT recipients as a prophylaxis.^34^ Unsurprisingly, resistance mutations to GCV and VGCV occur most often in the HCMV genes encoding UL97 and UL54.^34^ GCV dose is limited by cytotoxicity which can cause neutropenia and thrombocytopenia and caused temporary and permanent infertility in animal studies.^34^ This GCV-mediated neutropenia can lead to increased mortality from bacterial and fungal infections,^35^ and most worryingly, the myelosuppressive effects of GCV likely interfere with reconstitution of the immune system after HSCT.^36^ Despite these complications, GCV and VGCV are very effective and still the gold standard antiviral treatments, which keep HCMV disease incidence below 10%. To mitigate the negative side effects, GCV is routinely given for 100 days after SOT to limit neutropenia and nephrotoxicity.^37^ However, most cases of HCMV disease occur after this 100 days of treatment (late-stage HCMV disease). This is partly due to the virostatic nature of GCV and partly because these antivirals do not target latently infected cells. One proposed solution to this is to extend VGCV treatment to 200 days with one study suggesting that this regimen significantly reduced viraemia with no increase in adverse events.^37^ Other alternatives to prophylactic treatment include monitoring patients for HCMV viraemia or viral genome by PCR and preemptively treating with GCV, which shows similar efficacy to universal prophylactic treatment. This management strategy is currently used for patients after allo-HSCT as GCV, VGCV and foscarnet (discussed later) are too toxic to give prophylactically to HSCT recipients.^38^

## Second line drugs against HCMV

In addition to GCV are the second-line drugs cidofovir (CDV) and foscarnet which both preferentially inhibit the viral DNA polymerase over cellular polymerases.^34^ Acyclovir is also approved for the prevention of HCMV infection in the European Union. However, all these antivirals target the viral DNA polymerase, UL54, and so resistance mutations to GCV often lead to various levels of cross-resistance to other available antivirals.^34^ Foscarnet is also known to be nephrotoxic; it can result in metabolic changes as well as cardiac arrhythmias and genital ulcerations.^39^ Again, CDV is nephrotoxic and causes neutropenia, metabolic acidosis and ocular hypotony.^39^ Promisingly, brincidofovir is a prodrug of CDV which is conjugated to a lipid and is released intracellularly to improve drug efficacy; however, it recently failed to significantly reduce HCMV infection in renal transplant and HSCT recipients.^40^^,^^41^ Given these complications, there is still a desperate need for new antivirals against HCMV, particularly ones that do not target the DNA polymerase UL54.

Off-label leflunomide (an immunosuppressive normally used to treat rheumatoid arthritis) has anti-HCMV activity as a result of its ability to inhibit tegument formation.^42^ Although this drug is used in cases of GCV-resistant HCMV infection, results have been mixed.^43^

## Letermovir is a newly approved anti-HCMV antiviral which inhibits the viral terminase complex

The viral terminase complex is highly specific to herpesviruses; no cellular protein carrying out its function has been identified in mammalian cells, making this viral complex a good target for antivirals. A trimer of the proteins pUL51/pUL56/pUL89, the terminase complex binds the newly synthesised HCMV genome and the HCMV pro-capsid and uses ATPase activity to translocate the DNA into the capsid.^40^ Compounds that target the terminase complex have shown great anti-HCMV efficacy, including a hydroxypyridonecarboxylic acid compound, which inhibited pUL89 at low concentrations, and other compounds which had been discovered over the last two decades, but whose efficacy was undermined by poor bioavailability.^40^

Letermovir (Prevymis©), a novel anti-HCMV antiviral, is a quinazoline, targets pUL56 of the HCMV terminase complex and has an EC50 over 400-fold lower than GCV.^40^ Letermovir does appear to interact with the immunosuppressants given to patients after HSCT, cyclosporin A or tacrolimus, increasing their exposure, while letermovir also increased cyclosporin A pharmacokinetics.^40^ Importantly, letermovir does not seem to have antagonistic effects when combined with currently approved HCMV antivirals *in vitro*, raising the possibility that combinations of these antivirals could be used to treat HCMV.^40^ As renal and hepatic impairment affects letermovir pharmacokinetics, increasing exposure, this may affect its use in kidney and liver transplant recipients.^40^

Letermovir, licensed by AiCuris and Merck, has recently been approved for the prophylactic treatment of HCMV disease in HSCT recipients in the USA, Canada and the European Union. This is after successful Phase III clinical trials (www.accessdata.fda.gov, Reference ID 4179078).^40^ Letermovir was not recommended by the National Institute for Health and Care Excellence (NICE) for prophylactic use, with treatment beginning any day up to 28 days after HSCT and continuing for 100 days after initiation. This was due to the cost effectiveness estimate above £20 000 per quality adjusted life year (QALY), uncertainty over whether letermovir reduces HCMV mortality and questions about the generalisability of trial data to standard NHS practice.^44^ The appraisal committee did, however, recognise the need for new treatments that could reduce the need for preemptive therapies and also recognised that the cost effectiveness estimate per QALY would be affected by small changes in letermovir’s mortality benefit, and so was likely underestimated.^44^ A lower cost effectiveness estimate for letermovir, which could permit NICE approval, might be possible if more trial data is generated, particularly using patients in the UK and which analyses all-cause mortality and health-related quality of life at 48 weeks after allo-HSCT.

## Maribavir is a promising new antiviral against the viral UL97 kinase

Maribavir (MBV), developed by ViroPharma, is another promising anti-HCMV compound, which is administered orally and targets the viral kinase UL97.^45^ UL97 is required for correct formation of the viral tegument, formation of the viral assembly complex within the cell and virus release.^46^ However, co-administration of both MBV and GCV is not advised as the former is an inhibitor of the UL97 enzyme required for anabolism of the latter.^47^ One benefit to MBV is that it shows reduced haematotoxicity and nephrotoxicity compared to GCV and VGCV and so could eventually replace these older compounds.^48^ A randomized phase II trial comparing preemptive treatment of adult transplant recipients concluded that MBV has similar efficacy to VGCV at 400–1200-mg doses per day.^49^ However, a Phase III trial concluded that there was no difference between MBV (at 100 mg daily) and placebo, as a prophylaxis for HCMV disease following HSCT.^50^ This trial administered MBV at the lowest of three doses that were used in a preceding Phase II clinical trial (100, 200 and 400 mg). Repeating this trial using the highest MBV dose (400 mg daily), which is the predicted dose for adequate IC50, could prove to be clinically beneficial.^51^ Despite this setback, MBV is also being tested as a treatment for HCMV disease; one Phase III trial is testing MBV (200 mg daily) for transplant recipients with HCMV that already show resistance to GCV, CDV or foscarnet (clinicaltrials.gov, Identifier: NCT02931539).

## Large molecule therapeutics against HCMV

Passive immunisation with HCMV immunoglobulin (CMVIG) is approved by the US FDA for prophylactic anti-HCMV treatment in conjunction with GCV in high-risk lung transplant recipients.^52^ HCMV immunoglobulin therapy has also shown success in cardiothoracic transplant recipients^52^ and may be especially effective for those with complications such as GCV resistance, poor tolerance of GCV^52^ or hypogammaglobunaemia.^52^ One example of CMVIG efficacy was in a study where 15 asymptomatic heart transplant recipients, who had acute HCMV infection with no HCMV disease and low viral load, were given CMVIG preemptively without antivirals. Nine of these patients were negative for HCMV by PCR test after a single dose, and 14 were negative after two doses.^52^ Evidence is lacking to support CMVIG therapy to prevent congenital HCMV, and in a randomised trial, CMVIG did not significantly reduce congenital HCMV disease,^53^ but the treated group did report higher obstetrical adverse events.^53^ CMVIG is not recommended for routine use in pregnant women with primary HCMV infection.^19^

In addition to CMVIG therapy, a fusion toxin protein (FTP) has been developed with anti-HCMV activity. This protein, named F49A-FTP, consists of the soluble portion of fractalkine (CX3CL1), a signalling chemokine which controls inflammation and cell chemotaxis, genetically fused to the pseudomonas exotoxin (PE) A protein.^54^ The fractalkine moiety binds to the HCMV protein US28, a chemokine receptor which is expressed on the cell surface, and this binding leads to internalisation of both US28 and F49A-FTP. Once inside the cell, the PE portion of F49A-FTP is cleaved and inhibits translation, leading to cell death. F49A-FTP showed superior efficacy compared to GCV, in both infected fibroblasts and in a SCID-hu mouse model of HCMV disease.^54^ HCMV quickly became resistant to F49A-FTP, evolving mutations in the US28 gene to reduce FTP binding. As US28 is dispensable for HCMV replication, this suggests that F49A-FTP is unlikely to be used alone to treat HCMV disease, but could be used in combination with other antivirals to treat GCV-resistant HCMV. Indeed, F49A-FTP is virucidal, rather than virustatic, and so could be used to reduce viral loads in patients undergoing prophylactic treatment. The major concern with F49A-FTP treatment is likely to be off-target binding to the fractalkine receptor, CX3CR1, which is expressed at high levels on cells in the immune system and nervous system.^54^ F49A-FTP also showed efficacy in killing latently infected cells (discussed in the next section).

## Therapies against HCMV latency

HCMV latency is the maintenance of the viral genome in an infected cell which, depending on the latency model used, can be accompanied by the expression of a wide number of viral transcripts^55–57^ but importantly occurs in the absence of production of infectious virions.^3^ Similarly, by definition such latently infected cells have the potential to reactivate to lytic infection under specific stimuli.^3^ These stimuli include pro-inflammatory signals and myeloid differentiation.^3^ All current HCMV antiviral therapies target aspects of HCMV replication and virus production and are, therefore, ineffective against latently infected cells where HCMV does not actively replicate. Indeed, late-stage HCMV disease in transplant recipients likely occurs in part due to the reactivation of latent HCMV. HCMV can establish latent infection in early myeloid lineage cells, including CD34^+^ progenitor cells and CD14^+^ monocytes, the former of which comprise the major cell type present in HSCTs. Latently infected peripheral blood monocytes are also likely to be present in SOTs and, therefore, may also harbour virus which will not be targeted by VGCV treatment. Consequently, the reduction in latently infected cells in transplanted tissues could have far-reaching clinical benefits, and there has been significant recent interest in treatments against HCMV latency.^58^

Among these potential treatments is vincristine, a tubulin inhibitor which is used to treat a number of cancers. Vincristine is transported out of cells by the drug transporter multidrug resistance-associated protein 1 (MRP-1). However, the viral gene UL138 which is expressed during HCMV latency, downregulates MRP-1 making latently infected cells susceptible to vincristine toxicity.^59^ Vincristine was the first published molecule which could kill latently infected cells in culture,^59^ though the potential toxicity of vincristine may limit its clinical uses.

As discussed above, F49A-FTP binds the HCMV protein US28 and kills infected cells via a fused PE protein.^54^ US28 is also expressed by HCMV during latent infection,^60^^,^^61^ and this expression of US28 makes latently infected cells susceptible to killing by F49A-FTP.^61^ The major limitation of F49A-FTP is the emergence of resistance mutations in the US28 gene. However, this is less problematic during latent infection when virus replication does not occur. Given the unknown potential for off-target effects of F49A-FTP, one possibility could be to infuse transplanted tissues with F49A-FTP in order to clear infected cells before transplant.

In addition to directly virucidal therapeutics, two publications have proposed ‘kick and kill’ strategies to target the latent reservoir. This method to clear latently infected cells involves a treatment which forces the virus to at least partially reactivate from latency, resulting in expression of the viral major immediate early (IE) lytic proteins. These cells can then be detected and killed by the host immune system which is especially effective because a substantial proportion of circulating CD8^+^ cytotoxic T cells (CTLs) in healthy HCMV-seropositive carriers already recognise these viral IE antigens^62^ which are not normally expressed on latently infected cells. Treatment of latently infected cells with sodium valproate, an inhibitor of histone deacetylase 4 (HDAC4) and routinely used to treat epilepsy and bipolar disorder, causes changes in chromatin structure around the viral major IE promoter.^63^ This leads to transient aberrant expression of HCMV IE protein in the latently infected cell, which is then detected by the immune system allowing these cells to be killed.^64^ Alternatively, as the HCMV protein US28 is absolutely required for the maintenance of viral latency^61^^,^^66^, inhibition of US28 also leads to expression of HCMV immediate early protein in latently infected cells and their subsequent CTL-mediated killing.^65^

## Vaccines against HCMV are still in development

An HCMV vaccine was assigned in the highest priority category by the Institute of Medicine and the second highest priority target after HIV by the Centers for Disease Control.^66^ This is due to the recognised combination of widespread infection, large disease burden and limited applications for antiviral drugs and newer therapeutics.^66^ Such a vaccine could greatly reduce the risk of HCMV disease post-transplantation as well as decrease the rates of HCMV infection during pregnancy, thereby reducing rates of congenital HCMV transmission—as long as this vaccine elicited an immune response similar to that of natural immunity.^66^ In addition to acute HCMV disease, HCMV increases the risk of cardiovascular disease and all-cause mortality.^8^ Given the increased seroprevalence of HCMV in lower socioeconomic populations, such as African-Americans,^67^ an HCMV vaccine could help alleviate some of the observed socioeconomic disparities in health outcomes between populations.^67^

Analysis of the immune response to HCMV clearly indicates that a robust antibody and cellular response will be needed to confer protection.^68^ Although attempts to generate an HCMV vaccine date back to the 1970s, a successful vaccine candidate has yet to be developed, which suggests that HCMV requires a strong and specific immune response to confer protection against primary infection.^69^ Live attenuated virus vaccines, based on the laboratory-passaged Towne strain of HCMV (which has adapted to growth in laboratory fibroblasts), have a strong safety record and the virus is not shed from vaccinated individuals, suggesting that the virus does not cause a systemic infection.^69^ However, Towne virus vaccines have provided only weak protection against HCMV disease in renal transplant patients^70^^,^^71^, primary HCMV infection in seronegative women^72^ or protection against challenge with the lower-passage Toledo strain of HCMV.^73^ The most likely reason for this poor protection is that Towne virus has been passaged in the laboratory for too long and, hence, shows poor efficacy due to genetic differences between Towne and WT virus.^74^ Major genetic differences are seen between wild-type virus and Towne in the ULb’ region of the genome, which undergoes extensive deletions, rearrangement and mutations after serial passage in laboratory fibroblasts. The ULb’ region encodes multiple immune evasion proteins which interfere with NK cell recognition, such as UL135 (disruption of the immunological synapse), UL141 and UL142 (prevention of expression of NK cell activating ligands) as well as UL148 which disrupts CD58 expression, interfering with both NK cell as well as T cell recognition.^75^ Disruption of these genes would lead to faster clearing of Towne virus by the innate immune system. Additionally, genes required for the protein components of the pentameric complex (PC), mutate independently of ULb’. The PC is a protein pentamer that is present on the virion surface and is required for entry into epithelial cells, endothelial cells and leucocytes.^74^ As Towne virus has mutations in *UL130,* rendering it incapable of expressing the PC,^69^ it cannot induce an antibody response against the PC, which is known to be important for protection against WT virus infection.^69^^,^^76^

One solution to this problem is to recombine the high passage Towne laboratory strain with the lower passage, more wild type-like, Toledo virus to generate a chimera. This is still unable to establish systemic infection or latency but may be sufficiently similar to wild-type virus to provide protection against primary infection.^74^ These chimeras showed strong safety profiles and induced conventional CD8^+^ T cell responses, but showed differences in immunogenicity between chimeras.^74^ Unfortunately, DNA sequencing analysis showed that all four chimeras had disrupted copies of the *UL128* gene and so could not express WT PC.^77^ However, this sequencing data may provide useful information for understanding how genetic differences between attenuated viruses correlate with differences in immunogenicity between chimeras, which could inform future vaccine design.

HCMV dense bodies, which are produced by HCMV-infected cells *in vitro*, consist of viral tegument and glycoprotein-rich envelopes (including the immunodominant epitopes pp65 and gB), but lack viral capsids or viral DNA. These particles induce antibody and T cell responses, even without adjuvants and are, therefore, a potential vaccine candidate against HCMV.^78^ Whether they provide protection against virus, and whether these dense bodies can be generated in sufficient volumes (without contamination from live virus), remain to be answered.

## Subunit vaccines against HCMV have so far shown the highest protection against HCMV infection

More recently, recombinant subunit vaccines have been developed, based on the two immunodominant epitopes of HCMV: phosphoprotein (pp)65, a protein present in the virus tegument, and glycoprotein B (gB), which is expressed on the virus surface and is required for viral entry into cells.^74^ Immunisation against these two proteins has been delivered as vaccines using different methods including the solubilised proteins, DNA vaccines consisting of plasmids containing the genes encoding pp65 and gB, as well as viral replicon particles, which are replication-deficient virions that express high levels of encoded proteins and induce a strong immune response.^74^ A recent landmark vaccine consisted of recombinant HCMV gB with MF59 adjuvant; this showed 50% efficacy in protecting young mothers from HCMV infection in Phase II trials as well as protection against viraemia for R-D+ transplant recipients, the most efficacious results to date.^79^ This vaccine was also able to boost gB-specific antibody responses in seropositive women and so could help protect pregnant women from cCMV transmission caused by reinfection with a different strain of HCMV.^80^ Given the relatively low force of infection of HCMV, the gB/MF59 vaccine would likely have sufficient efficacy to substantially reduce the circulation of HCMV in the human population.^81^^,^^82^ Interestingly, despite this vaccine showing protection, antibodies from patients inoculated with this vaccine failed to neutralise HCMV in standard laboratory assays, which suggests that more detailed laboratory analyses may be needed for viruses such as HCMV which establish lifelong infection and likely transmit in a cell-to-cell-based manner.^83^

## It is still unclear what immune responses are needed to protect against HCMV infection

The observation that HCMV can superinfect an already persistently infected host^84^ suggests that the development of an effective HCMV vaccine will be difficult. Indeed, HCMV vaccines may only be effective at reducing the probability of HCMV infection in high-risk populations such as pregnant mothers and transplant recipients. It also remains unclear what aspects of anti-HCMV immunity provide protection in different scenarios.^74^ For example, evidence suggests that prior infection with HCMV reduces the risk of a secondary infection for pregnant mothers; however, HCMV IgG therapy does not provide similar protection^85^ despite early evidence that it may have been protective.^86^ Other studies have suggested that early antibody responses against the HCMV PC, maternal CD4^+^ and CD8^+^ T cell responses^85^ and low interferon relative responses to cytomegalovirus are associated with low likelihood of intrauterine transmission of the virus and all seem to correlate with protection.^74^^,^^85^ Finally, as stated already, the presence of neutralising antibodies may not be the best measure of vaccine efficacy^83^ and, indeed, the presence of non-neutralising antibodies may be counterproductive, for example by promoting HCMV translocation across the placenta via Fc receptor-rich trophoblasts, leading to increased risk of congenital infection.^87^

## Conclusion

HCMV is a significant pathogen, and we contend that the eradication of this virus would benefit humanity greatly. There is currently no vaccine against HCMV, but there are reasons to be hopeful: the relative success of the varicella-zoster virus (VZV) vaccine,^88^ advances in vaccines against HSV-2^89^ and a subunit vaccine which provided 50% protection against HCMV^79^ all suggest that a vaccine is possible. Due to the ubiquity and lifelong nature of HCMV infection, any vaccine against HCMV for the general population will likely take a very long time to eradicate the virus from the human population. Until this time, novel antivirals against HCMV are still desperately needed to treat HCMV disease. One such drug is letermovir, an inhibitor of the viral DNA packaging, while other inhibitors (against both HCMV lytic replication and HCMV latent infection) have shown great promise.
